# Preferences of diabetes patients and physicians: A feasibility study to identify the key indicators for appraisal of health care values

**DOI:** 10.1186/1477-7525-8-125

**Published:** 2010-11-04

**Authors:** Franz Porzsolt, Johannes Clouth, Marc Deutschmann, Hans-J Hippler

**Affiliations:** 1 Clinial Economics, University of Ulm, 89073 Ulm, Germany; 2 Lilly Deutschland GmbH, 61352 Bad Homburg, Germany; 3 BIK-MARPLAN Intermedia GmbH, 63065 Offenbach, Germany; 4 SRH University of Applied Sciences, 75365 Calw, Germany

## Abstract

**Background:**

Evidence-based medicine, the Institute of Medicine (IOM) and the German Institute for Quality and Efficiency in Health Care (IQWiG), support the inclusion of patients' preferences in health care decisions. In fact there are not many trials which include an assessment of patient's preferences. The aim of this study is to demonstrate that preferences of physicians and of patients can be assessed and that this information may be helpful for medical decision making.

**Method:**

One of the established methods for assessment of preferences is the conjoint analysis. Conjoint analysis, in combination with a computer assisted telephone interview (CATI), was used to collect data from 827 diabetes patients and 60 physicians, which describe the preferences expressed as levels of four factors in the management and outcome of the disease. The first factor described the main treatment effect (reduction of elevated Hb_A1c_, improved well-being, absence of side effects, and no limitations of daily life). The second factor described the effect on the body weight (gain, no change, reduction). The third factor analyzed the mode of application (linked to meals or flexible application). The fourth factor addressed the type of product (original brand or generic product). Utility values were scaled and normalized in a way that the sum of utility points across all levels is equal to the number of attributes (factors) times 100.

**Results:**

The preference weights confirm that the reduction of body weight is at least as important for patients - especially obese patients - and physicians as the reduction of an elevated Hb_A1c_. Original products were preferred by patients while general practitioners preferred generic products.

**Conclusion:**

Using the example of diabetes, the difference between patients' and physicians' preferences can be assessed. The use of a conjoint analysis in combination with CATI seems to be an effective approach for generation of data which are needed for policy and medical decision making in health care.

## Background

Evidence based medicine suggests the consideration of patient's preferences but preferences are rarely assessed in clinical trials. Reason for not considering preferences may be that most studies focus only the assessment but not yet the appraisal of treatment effects and that the assessment and appraisal of effects require different methods. Scientists can describe treatment effects (assessment). In addition to the description of observed effects it may also be important to record and describe the value of such effects i.e. what these effects mean to somebody. As an example, the reduction of body weight is usually higher valuated by women than by men. This step of evaluation, i.e. putting a value to a certain effect may be considered as appraisal. The separation of assessment and appraisal of a treatment - or of any other effect - may be rather important as decisions are generally based on values but not only on effects [1].

Effects may be observed under ideal, but possibly artificial conditions or under real world conditions. Trials which describe observed effects under ideal conditions (i.e., which describe efficacy), may be called explanatory trials [2-4]. These trials aim to identify a potentially causal relationship between the intervention and the observed outcome. Trials which describe effectiveness through observed effects under real world conditions may be called pragmatic trials [2-4]. We consider these trials to identify effects which can also be detected under real world conditions. Confounders such as co-morbidity, co-treatments, stress factors and interpersonal relationship influence the outcome and are therefore eliminated in efficacy trials but not in effectiveness studies. Efficacy and effectiveness are two extremes of a continuum. In fact, there is a whole spectrum of explanatory and pragmatic trials [5].

The second level of reporting is related to the appraisal of the effect of the health service. Appraisal means that an individual ascribes a value to the observed effect. Values are based on preferences, preferences can be measured and preferences should definitely be considered in health care decisions [6]. Appraisals should ideally be confined to studies which are completed under real word conditions. A possible sequence of reporting the effects and of their meanings is shown in Table 1. Two assessments which were made under ideal and real world conditions should precede the appraisals from various perspectives, e.g., from the perspective of patients or doctors (Table 1).

**Table 1:** Possible sequences for reporting the effects of health care services

	Level of assessment	Level of appraisal
**Experimental clinical trials conducted under ideal, but possibly artificial conditions**	1^st ^step Explanatory trial describing possible causal effects of an action under ideal conditions, i.e., describing the efficacy	Not useful

**Descriptive studies conducted under day-to-day, real world conditions**	2^nd ^step Pragmatic trial describing the effects of an action under real world conditions, i.e., describing the effectiveness	3^rd ^step Assessment of individual preferences under real world conditions, i.e. describing the value perceived by an individual

Two assessments under ideal (step 1) and real world conditions (step 2) at the level of assessment are followed by the appraisal of real world results (step 3) from various perspectives. As the available information is growing from step 1 to step 3, it is justified to value health care services the higher the more steps of this sequence were completed. Desired effects which can be detected only under ideal conditions of a clinical trial, but not under real world conditions will be valued lower than desired effects which can be detected also under real world conditions.

Hypotheses can be tested under ideal conditions. It is more difficult if not impossible to test hypotheses by data which were recorded under real world conditions [7]. The appraisal of health care services, i.e., the description of the value or benefit or utility of services, is difficult to falsify because these appraisals depend on individuals' preferences [8]. The validity of the methods used to describe the value, benefit, or utility of a health care service, such as Time-Trade-Off, Standard Gamble, and Quality Adjusted Life Years, is discussed controversially because these methods include the preferences of the raters and require assumptions which are sometimes not met in real world conditions [7,9,10], like in older patients with diabetes [11].

We have recently addressed the problem of different preferences of health care providers and health care users [12] by comparing patients' decisions with the recommendations of international guidelines for neo-adjuvant or adjuvant radiotherapy in the treatment of colorectal cancer. Although the treatment decisions (with or without radiotherapy) of both patients and professionals were based in our experiment on the same set of clinical trials, 85% of the patients refused the radiotherapy which was recommended in the guidelines. Survival was the same with and without radiotherapy, but fecal incontinence, a functional indicator, was considerably less frequent in the group without radiotherapy, while the reduction of the tumor size, i.e., a structural indicator, was more frequent in the group with radiotherapy. This example shows that health care providers and health care users express different preferences when they are confronted with identical information and are asked to decide according to their preferences. Therefore, preferences of both doctors and patients should be carefully analyzed when policy or clinical decisions are made.

The conjoint analysis is a well established method to identify preferences. This method was used in the UK, the Netherlands and the USA in several health care projects [13-17]. The aim of this paper is to identify the factors which are important for treatment decisions of diabetes type I and II in Germany and to compare the preferences of patients and doctors in this setting with policy decisions.

## Methods

### Selection of the target population

A sample of 1006 diabetic patients, aged 14 years or older, was identified from a previous general survey on 27,000 German households. Of these 1006 diabetes patients, 827 agreed to and were able to complete a computer assisted telephone interview (CATI). Part of this interview was a conjoint measurement module which included the four factors which were identified in the focus group.

### Identification of key factors and factor levels for the conjoint measurement procedure

To identify the important aspects of diabetes treatment for patients its outcomes were discussed in a focus group of ten diabetes patients. This focus group suggested four important factors for patients' decisions in the management of type 1 or type 2 diabetes. Two of the four factors were related to the effects of treatment, i.e., the main treatment effect and the effect of treatment on body weight. Two other factors were related to the mode of application and the type of product. These factors and the factor levels were used for the following conjoint procedure.

### Four steps to complete the conjoint measurement

The participants of the study had to complete four steps of the conjoint measurement to describe their preferences for a particular treatment. Each treatment was characterized by four factors. Within each factor, two to four factor levels could be selected. The four factors and the factor levels are shown in Table 2.

**Table 2:** Factor and factor levels as ranked by patients.

Factors	Factor levels	Weights of factor levels				
		All patients	Normal body weight	Mild over-weight	Adipositas I	Adipositas II+III
**Main treatment effect**	Reduction of elevated Hb_A1c_	48.4	48.9	47.6	49.7	44.7
	Improved well-being	37.5	34.7	35.6	40.4	40.5
	Absence of side effects	43.0	43.6	44.6	43.5	37.2
	No limitations of daily life	41.3	40.6	42.8	40.7	36.0

**Effect on body weight**	Weight gain	15.7	20.7	15.0	12.6	11.4
	No change	**55.8**	**65.8**	**55.7**	50.1	56.3
	Weight loss	54.9	36.5	53.8	**65.0**	**76.2**

**Mode of application**	Flexible time of application	30.4	29.2	32.2	29.8	28.5
	Application linked to meals	22.6	26.9	21.9	22.2	20.7

**Type of product**	Original product	36.2	37.3	36.9	33.2	35.0
	Generic product	14.3	15.8	13.9	12.8	13.5

Left side: Factors and factor levels which had to be ranked by the study participants. Right side: The weight of factor levels in the total patient population (n = 827) and in subpopulations of patients with normal body weight (22.6%), mild overweight (40.4%), obesity type I (25.8%) and obesity type II+III (11.2%) is shown. Differences in preferences among patient groups are highlighted.

First, participants were asked to rank the offered levels for each of the four factors. Second, several pairs of factor levels were presented to the participants to assess the weight of the factors. For that, the participants had to express the importance (from absolutely important to not important at all) they considered to the difference of two particular levels, i.e., to a decrease of body weight compared to an increase of body weight. Third, virtual pairs of drugs were created by combining different levels of three factors (e.g., option "A": generic drug, causing weight gain, flexible application or alternatively option "B": original drug, causing weight loss, application linked to meals).

The participants had to express their preference on a four item scale (strongly prefer "A", prefer "A", prefer "B", strongly prefer "B") for one of these options. Fourth, to confirm the validity of the calculated result, the participants were asked to describe the probability of using a virtual drug which was characterized by selected levels of the four levels (e.g., causing weight loss, reducing elevated Hb_A1c_, flexible time of application, original drug).

As the number of all possible combinations of factor levels is too high to be tested, the ideal combinations of factor levels were based on the responses to the preceding questions.

### Estimation of weights of factor levels

The data collection, as well as the estimation of utility weights, was done with the Adaptive Conjoint Analysis (ACA) software 1997 (Sawtooth Software, Inc., Sequim, Washington, USA). Like the most established approaches in conjoint analysis the ACA is based on a main-effects model. Due to the exclusion of attribute interactions, measuring of utilities for attributes takes place in a standard-all-else-equal context. Utility values were scaled and normalized by this method in such a way that the sum of utility points across all levels is equal to the number of attributes (factors) times 100. As there are four attributes in our model (main treatment effect, effect on body weight, mode of application and product type) the total amount of weight-points are 400. Depending on the reported preferences during the interview, these 400 points were itemized by established multiple regression analysis over the 11 factor levels in order to calculate utility values for all levels for each respondent by least square estimation. Finally, average utility weights were calculated and compared for different subpopulations of patients or their physicians, respectively.

## Results

### Characteristics of the patients and physicians

The telephone interview was completed by 827 patients, 46.9% of whom were male. Of these patients, 21% were aged 14-29, 5.7% were aged 30-49, and 92.3% were aged over 49. In 59% of diabetes patients, the annual net household income was below € 20.000, in 30% of patients, the annual net household income was between € 20.000 and € 30.000, and 11% of patient households had annual net income higher than € 30.000. The average annual net income of all households in Germany is € 33.700.-

Type 1 diabetes was diagnosed in 9% of patients, type 2 diabetes was diagnosed in 89% of patients, and 2% of patients couldn't be allocated. The sex distribution was similar in type 1 and type 2 patients. Obesity type II and III were observed in 5% of patients with type 1 diabetes, but was found in 12% of patients with type 2 diabetes. No obesity was observed in 51% of patients with type 1 and in 20% of patients with type 2 diabetes. The diabetes was treated with oral medication in 47% of patients; 29% of patients were treated with insulin, 14% of patients were treated with combined oral and insulin therapies, and 11% of patients did not receive either oral or insulin treatment. Diabetes was known for 1-5 years in 38% of patients, for 6-10 years in 25% of patients, for 11-15 years in 13% of patients, and for 15+ years in 23% of patients.

To prevent a possible selection bias, the patient characteristics of the total sample of the selected diabetic patients (n = 1006) were compared with those of the subgroup of patients who agreed to and were able to complete the conjoint measurement questions (n = 827). The maximal absolute difference in the reported patient characteristics was 0.3% which renders a bias non-responders rather unlikely (data not shown).

Sixty physicians, including 30 general practitioners and 30 diabetes specialists were also included in the study. Their average number of years of professional experience was 22.5 and 22.9 years, respectively. The general practitioners had an average of 171 diabetes patients in their practices and the diabetes specialists had an average of 331 diabetes patients in theirs.

### Weight of factor levels

The weights of the levels of the four factors based on assessments in 827 patients were calculated for the entire group, as well as for subgroups, according to the type of diabetes, gender, age, treatment, body weight, and for combinations of these characteristics. A selection of these data is included in Table 2 where the weights of factor levels according to body weight are shown.

This database offers the possibility to compare the preferences within one group of patients or among groups of patients. Patients consistently valued the main treatment effects higher than the modes of application and weight loss was more important for obese patients than for non-obese patients (Table 2).

Data assessed in 60 physicians are shown in Table 3. In the physician group, the main treatment effects were not always valued higher than the modes of application as in the patient group. It is also shown that general practitioners clearly preferred generic products over original products. This difference was not seen in diabetes specialists.

**Table 3:** Factor and factor levels as ranked by physicians.

Factors	Factor levels	Weights of factor levels		
		
		All physicians	General practitioners	Diabetes specialists
**Main treat-ment effect**	Reduction of elevated Hb_A1c_	61.3	63.7	58.8
	Improved well being	43.8	53.7	33.9
	Absence of side effects	30.0	30.6	29.4
	No limitations of daily life	28.4	28.5	28.2

**Effect on body weight**	Weight gain	7.1	8.6	5.6
	No change	57.9	54.8	60.9
	Weight loss	71.3	68.4	74.2

**Mode of application**	Flexible time of application	16.0	12.5	19.4
	Application linked to meals	29.9	28.9	30.8

**Type of product**	Original product	17.1	**3.7**	**30.6**
	Generic product	37.3	**46.5**	**28.2**

Table 3. Factors and factor levels ranked by the general practitioners and diabetes specialists are shown. Differences in preferences are highlighted.

**Figure 1:**
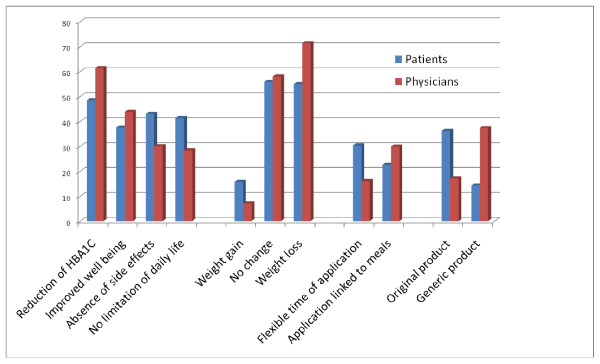
**Factor level analysis**. Factor levels of the four factors, main treatment effect, effect on body weight, mode of application, and type of product assessed in 827 diabetes patients and 60 physicians are shown.

The comparison of patients and physician assessments demonstrated that the reduction of Hb_A1c _and the reduction of body weight were more important for physicians than for patients. Patients clearly preferred original products, while physicians generally seemed to prefer generic products (Figure 1). The more detailed analysis in Table 2 demonstrates that the physicians' preference of generic products was confined to general practitioners.

## Discussion

There is an increased awareness of the need to involve patients in policy and clinical decision making as psychological factors like risk aversion [18] and perception of information are important variables which influence decisions, as well as final outcomes [19]. This applies especially to patients with chronic conditions, like diabetes mellitus [20,21]. These psychological factors are expressed as preferences which may be assessed by a conjoint analysis.

This study investigated the feasibility of a conjoint measurement for assessment of preferences in diabetic patients in Germany. It should be emphasized that our study refers to patient preferences but not to treatment decisions. Patient preferences may play an important role in the trade off of different properties of a therapy but not all therapies may cover the patients' preferences. The obtained information is rather important as the consideration of patients' preferences was requested as part of evidence-based decisions [6]. A second aspect of preferences is related to the selection of the appropriate study endpoints for description of patients' benefit. These endpoints should consider the patients' preferences, in addition to medical and economic aspects. The obvious difference between physicians' and patients' preferences has been demonstrated in this and other [12] studies. These differences can lead to conflicting result as exemplified in the paradox outcome of treating a schizophrenic patient (personal communication). The added value of such a treatment may be questionable when the patient realizes after successful treatment that he or she has neither a job, nor money, nor a partner. From the physician's point of view, the symptoms of the disease may have been treated successfully. From the patient's point of view, it remains unclear if the optimal quality of life could be achieved just by reduction of the symptoms of the disease. A corresponding result was seen in our study. According to the assessed preferences of both patients and physicians, weight loss is at least as important as the reduction of an elevated HB_A1c _(Tables 2 and 3). This means that weight loss and reduction of an elevated HB_A1c _may be used as equivalent endpoints in pragmatic trials, which is not really the case. We expected that the focus groups would include mortality, morbidity and functional status as important outcomes. As none of these items were mentioned by the focus groups it seems that patients' short term goals and goals that are frequently discussed at consultations are more important than remote health goals and less frequently discussed aspects.

Our study also demonstrated that physicians and patients prefer different types of products. Patients prefer original brands, while general practitioners - but not diabetes specialists - prefer generic products. This difference in preferences is explained by policy decisions in Germany. Practitioners who are under budget control and prescribe most of the treatments prefer to prescribe the less expensive products. Specialists who mainly recommend, but do not have to prescribe the treatments, expressed no preference for original or generic brands. The patients' preference for the original brand is most likely explained by the initial use of original products and the discomfort associated with the change of treatment from the original to generic products. This change is usually induced by physicians who have to consider the cost-effectiveness. Unfortunately, cost-effectiveness analysis can include only a limited number of aspects but may be improved if the factors which are important for decisions have been identified in advance. Health services will improve if the main patients' problems are addressed and if appropriate answers can be given to solve these problems. Accordingly, the German Advisory Council on the Assessment of Developments in the Health Care System (Sachverständigenrat) recommended in its 2007 report to consider scientific methods for selection of the appropriate endpoints [22]. The conjoint analysis may be a useful tool for identifying the patient's problems and preferences.

It is difficult to predict if the results of this study will also apply to patients in other cultures because the identification process in the focus group was based on a rather small sample and the method for selecting the factors was not too robust as a large number of tests was completed but the results of only some tests were interesting enough to be reported. In fact, this study did not test a hypothesis but rather generated a hypothesis. These weak points of the study may be improved in subsequent trials.

The conjoint analysis is not the only method to make preference-based decisions. Other methods such as the discrete choice analysis, which involves choices between two or more discrete alternatives, or the rational choice theory, may also be a valuable method to support health policy decisions. It should be remembered that all of these methods are based on individual decisions which cannot be falsified. This does not mean that data which cannot be falsified are less valuable than data that can. The two types of data just represent two types of decision making. We recommended considering both types of data for policy decisions in health care.

The report of the Institute of Medicine on Comparative Effectiveness Research [23] requested that patients' preferences wee included in health care decisions. This request supports our model, which is based on the levels of assessment and appraisal as shown in Table 1. The appraisal of health care services presumes that preferences can be measured and can be made available to the policy and decision makers. The CATI technique seems to guarantee the fast and effective generation of these data. In our study, 827 of 1006 eligible patients (82%) completed the telephone interview. A biased selection of the 827 patients is rather unlikely, as the patient characteristics of this population were very similar to the characteristics of all eligible patients (data not shown). The costs of this technique have to be balanced against the fast decision that can be made and the consequences which can be derived for the patients, health care providers, and manufacturers. The inclusion of patients' preferences in the process of policy and clinical decision making reflects the new area of evaluation and interpretation in health care. The methods used in this report may become important tools in this new area.

## Conclusion

There is sufficient evidence that conjoint analysis is an efficient method to analyze data which are needed for evidence-based decision making in health care. Important aspects for policy decisions in diabetes mellitus from physicians', as well as patients', point of view are the reduction of an elevated HB_A1c_, as well as the reduction of obesity. Original brands are preferred by patients, while generic brands are preferred by general practitioners. This approach is interesting for future attempts where patients' preferences will have to be included in health policy and clinical decisions.

## List of abbreviations

ACA: adaptive Conjoint Analysis (ACA); CATI: computer assisted telephone interview; Hb_A1c_: hemoglobin A subtype 1c

## Competing interests

Franz Porzsolt is a consultant of Lilly Deutschland GmbH, initiator of the Wilsede Workshop for Outcomes Research and member of the jury of the Quality of Life Award. The Wilsede Workshop as well as the Quality of Life Award are sponsored by Lilly Deutschland GmbH. Johannes Clouth is manager of health economics at Lilly Deutschland GmbH. Marc Deutschmann and Hans-J. Hippler are working with BIK-MARPLAN Intermedia GmbH.

## Authors' contributions

FP developed the concept of the publication and wrote the draft of the manuscript. JC initiated the study and developed the details of the study together with HJH. MD and HJH completed the study. All authors participated in the discussion and interpretation of the results as well as in the preparation of the final manuscript which was also approved by all authors.
